# Song: What Makes You Albumin (to the tune of “What Makes You Beautiful”*)

**DOI:** 10.1002/bmb.70028

**Published:** 2025-12-23

**Authors:** Seshadri Reddy Varikasuvu, Derek T. McLachlin

**Affiliations:** ^1^ Department of Biochemistry All India Institute of Medical Sciences (AIIMS) Deoghar Jharkhand India; ^2^ Department of Biochemistry Western University London Ontario Canada

**Keywords:** albumin, humor, music, parody

1

Made in liver, I know what you're for
Binding hormones, drugs, lipids, and more
Half the protein the plasma's seen
You have disulfide bonds from your cysteines
Every analyte in the blood can feel it
Every tissue but you

Baby you buffer pH like nobody else
Maintain oncotic pressure so that I stay well
When oxidative stress comes, you protect my health
You don't know
You don't know you're albumin

If you saw what you transport for me
You'd understand your role in physiology
Right now I'm charting serum biochemistry
You don't know
You don't know you're albumin
That's what makes you albumin

So c‐come on, you fold up strong
To prove I'm right, I put you in this song
You warn us of ischemia
When oxidative conditions are rough
Every analyte in the blood can feel it
Every tissue but you

Baby you buffer pH like nobody else
Maintain oncotic pressure so that I stay well
When oxidative stress comes, you protect my health
You don't know
You don't know you're albumin

If you knew you scavenge ROS for me
You'd understand why you're so vital clinically
Right now I'm tracking oxy‐reactivity
You don't know
You don't know you're albumin
That's what makes you albumin

Baby you quench free radicals like no one else
You escort bilirubin and drugs with stealth
In hypoalbuminemia, you affect our health
You don't know
You don't know you're albumin

If you saw how you change fluid flow
You'd understand why edema comes when you're low
Right now I'm writing this song just to let you know
You don't know
You don't know you're albumin
You don't know you're albumin
That's what makes you albumin

## Conflicts of Interest

The authors declare no conflicts of interest.

## Data Availability

Data sharing not applicable to this article as no datasets were generated or analysed during the current study.

